# Delayed Immune Response Upon Injury in Diabetic Wounds Impedes Healing

**DOI:** 10.1002/iid3.70142

**Published:** 2025-01-31

**Authors:** Priyanka Dhanraj, Kiara Boodhoo, Mari van de Vyver

**Affiliations:** ^1^ Experimental Medicine Research Group, Department of Medicine, Faculty of Medicine and Health Sciences Stellenbosch University Cape Town Western Cape South Africa

**Keywords:** diabetes, eicosanoids, inflammation, MPO, TNFa, wound healing

## Abstract

**Background:**

Chronic wounds are a severe complication of diabetes. Dysregulated inflammatory signalling is thought to underly the poor healing outcomes. Yet, there is little information available on the acute response following injury and its impact on healing.

**Methods:**

Using a murine full thickness excisional wound model, the current study therefore assessed the expression of pro‐inflammatory and pro‐resolving lipid mediators during the early stages post injury in acute and diabetic wounds and compared the timeframe for transitioning through the phases of healing. Tissue eicosanoid (LTB4, PGE2, TxA2, MaR1, RvE1, RvD1, PD) and MMP‐9 levels were assessed at 6 h post wounding using ELISAs. Wound closure, healing dynamics (histology), cellular infiltration and MPO, TNF‐α expression (IHC) were assessed at 6 h, day2, day7 post wounding.

**Results:**

Eicosanoid expression did not differ between groups (LTB4 24–125 pg/mL, PGE2 63–177 pg/mL, TxA2 529–1184 pg/mL, MaR1 365–2052 pg/mL, RvE1 43–1157 pg/mL, RvD1 1.5–69 pg/mL, PD1 11.5–4.9 ng/mL). An inverse relationship (*p* < 0.05) between MMP‐9 and eicosanoids were however only evident in acute and not in diabetic wounds. Diminished cellular infiltration (x5 fold) (*p* < 0.05) in diabetic wounds coincided with a significant delay in the expression of TNF‐α (pro‐inflammatory cytokine) and MPO (neutrophil marker). A significant difference in the expression of TNF‐α (C 1.8 ± 0.6; DM 0.7 ± 0.1 MFI) and MPO (C 4.9 ± 1.9; DM 0.9 ± 0.4 MFI) (*p* < 0.05) was observed as early as 6 h post wounding, with histology parameters supporting the notion that the onset of the acute inflammatory response is delayed in diabetic wounds.

**Conclusion:**

These observations imply that the immune cells are unresponsive to the initial eicosanoid expression in the diabetic wound tissue.

## Graphical abstract

Dysregulated inflammatory signaling is thought to underlie the poor healing outcomes. Yet, there is little information available on the acute response following injury and its impact on healing. This study supports the notion that the onset of the acute inflammatory response is delayed in diabetic wounds

## 1 Introduction

Diabetic foot ulcers (DFUs) are common and severe complications of diabetes mellitus (DM) with healing times ranging between 12 and 62 weeks [1, 2, 3]. The prevalence of DM is ever‐growing, affecting 537 million people, with obesity‐associated type 2 diabetes (T2DM) accounting for most cases [4, 5]. DFUs occur in 4–10% of patients living with DM, with a lifetime risk of 25% and a 20–80% recurrence rate [2, 6, 7]. These patients often require hospitalization and in severe cases result in lower limb amputation [8]. Non‐healing wounds not only impacts the individuals' quality of life but also remains a major unsolved health crises and economic burden [9]. Necessitating an improved understanding of the pathological changes occurring at tissue level to inform the development of regenerative strategies.

Wound healing is a carefully orchestrated series of events, to contain and eliminate invading pathogens, remove dead cells and rebuild tissue and vascular networks. In normal circumstances, this process proceeds naturally to restore homeostasis and tissue function through four phases namely, haemostasis, inflammation, proliferation and remodelling [10, 11]. Several lipid mediators (eicosanoids) such as prostanoids, 5‐lipoxygenase products and specialized pro‐resolving mediators play a role in facilitating tissue regeneration [12]. The prostanoids such as prostaglandin E2 (PGE2) and thromboxane A2 (TxA2) is produced by cyclooxygenase (COX) enzymes from arachidonic acid following breakdown of membrane phospholipids by phospholipase A2 (PLA2) upon injury. These lipid mediators have diverse functions, which includes initiation of the acute inflammatory response following tissue damage [12]. TxA2 plays an important role in vasoconstriction and stimulates platelet activation/aggregation during the hemostasis phase [13], whereas PGE2 plays a role in vasorelaxation to increase blood flow and hyperpermeability, plasma leakage, neutrophil recruitment and potentiates immune cell mediated cytokine signalling [14, 15]. Similarly, leukotriene B4 (LTB4) which is a product of 5‐lipoxygenase also stimulates the production of pro‐inflammatory cytokines and promotes the chemotaxis of immune cells (neutrophils, monocytes) into the injured area [16]. Neutrophils are the first immune cells recruited to the wound site and are most prominent 24–72 h post‐injury. Neutrophils remove cellular debris and microorganisms through phagocytosis, the release of granules containing antimicrobial molecules (myeloperoxidase (MPO)), the release of reactive oxygen and/or reactive nitrogen species (ROS/RNS), and through the formation of neutrophil extracellular traps (NETs) [11, 17, 18]. Neutrophils also secrete various inflammatory cytokines, which recruits additional neutrophils, as well as phagocytic monocytes/macrophages, and T cells [19, 20]. This pro‐inflammatory response is essential in ensuring the removal of debris and invading pathogens, as well as to prepare the wound for subsequent regeneration. This is followed by a resolution phase with pro‐resolving lipid mediators, derived from poly unsaturated fatty acids (PUFA), facilitating the transition into the proliferative stage of healing through macrophage activity. These factors attenuate LTB4 signals to regulate immune cell migration and cytokine production, promotes re‐epithelization, fibroblast migration, collagen deposition and nerve fibre growth [12]. A direct comparison of the healing dynamics and proteome of acute and diabetic wounds in the early versus later stages post wounding has previously demonstrated that dysregulation of several proteins during the first 2 days post injury was responsible for the delayed transition into the proliferative phase of healing [21]. This observation highlighted the need for understanding the early stages of wound healing, yet in patient‐based studies, the pathogenesis of chronic diabetic wounds has largely been investigated in deteriorating wounds which have been open for weeks and are often infected.

The pathophysiology of non‐healing diabetic wounds is complex [3, 22]. The hyperglycaemic environment and its accompanying microenvironmental changes (excess ROS, accumulation of advanced glycation end products (AGEs) and metabolic inflammation) leads to neuropathy, microvascular disease, ischemia and atherosclerosis, all of which contribute to the formation of ulcers and the inability of these wounds to progress through the phases of healing [3, 22, 23]. Recent studies have emphasized the role of metabolic inflammation and immune senescence in diabetic wounds [17]. Chronic low‐grade systemic inflammation ‘*Metabolic inflammation’* is largely driven by hyperglycaemia, adipose tissue expansion, oxidative stress, AGE accumulation, and promotes immune senescence [17, 24]. Several studies have since illustrated that immune senescence in DM is associated with impaired immune cell recruitment and functioning [17] resulting in an inadequate acute inflammatory response upon tissue injury. Yet, there is little to no information available on the spatial dynamics and eicosanoid dysregulation during the first few hours post wounding and its impact on the progression of healing. The current study, therefore, investigated the expression of pro‐inflammatory and pro‐resolving mediators during the early stages post injury and compared the timeframe for transitioning through the phases of healing on histological level in acute and diabetic wounds.

## 2 Materials and Methods

### 2.1 Murine Diabetic Wound Model: Ethical Considerations

This study was performed on 10–12 week old male B6. Cg‐Lep^ob^/J; ob/ob (obese diabetic, body weight > 40 g; blood glucose > 16 mmol/L) (*n* = 18; *n* = 6 per time point) and wild type C57BL/6 J mice (healthy control, body weight < 30 g; blood glucose < 11 mmol/L) (*n* = 18; *n* = 6 per time point) (Jackson Laboratory, ME, USA). All laboratory animals were specific pathogen free (SPF). Ethical approval was obtained from the Stellenbosch University research ethics committee for animal care and use (ACU:REC) (Ethics approval #SU‐ACUD17‐00016). The animals were housed under standard conditions at the animal research facility at the Faculty of Medicine & Health Sciences, Stellenbosch University, for the duration of the study. All procedures complied with the South African National Standards (10386:2008) and the Veterinary and Para‐Veterinary Professions Act, 1982 and was performed by trained para‐veterinary personnel. The animals were wounded (day 0) and wound healing was monitored for a period of 7 days. Animal welfare was closely monitored with behaviour and appearance noted twice daily for the duration of the study.

### 2.2 Induction of Full Thickness Excisional Wounds

Full thickness excisional wounds were induced using an established protocol. Once the animals were anesthetised using isofluorane gas (3% induction, 2% maintenance) (Safeline Pharmaceuticals), the dorsal hair of the animals were shaved, and the skin prepped with povidone‐iodine (Munipharma). At the base of the skull and on either side of the midline, two identical bilateral full‐thickness skin excisions ( ± 5 mm diameter) were made by applying outward retraction of the skin using sterile forceps and cutting the skin using sharp surgical scissors. The underlying *panniculus carnosus* muscle was removed to generate a full thickness wound. A diabetic wound was induced as previously described [21]. Pain management consisted of injecting a local anaesthetic, lignocaine (2%) (7 mg/kg) (Bodene, Port Elizabeth, South Africa) subcutaneously post wounding as well as administering 300 mg/kg acetaminophen (GSK, Cape Town, South Africa) to the drinking water for a period of 3 days. The wounds were dressed with a vapour‐permeable PU film (Hydro‐film, Baden‐ Württemberg, Germany) and secured with surgical tape. After recovery from anaesthesia, the animals were housed individually under standard conditions, with ad libitum access to chow (Rat and Mouse Breeder Feed, Animal Specialties, Pty Ltd., Klapmuts, SA) and drinking water. At each time point (0 h, 6 h, day 2, and day 7), macroscopic images of the wounds were digitally captured to calculate the change in superficial wound surface area (% wound closure) using ImageJ software 1.52 v, Java (http://imagej.nih.gov/ij). At the respective end‐time points, the animals were euthanised (anesthetized via an open system inhalation chamber using Isoflurane gas followed by an overdose of Halothane (B53M11A, Safeline Pharmaceuticals, South Africa)) and the wound tissue including a 5 mm margin excised (6 h, day 2, and day 7). Tissue samples were divided with one part being fixed in 10% neutral buffered formalin (NBF) solution and embedded in paraffin wax for histological analysis. The other part was snap frozen in liquid nitrogen (N_2_) and stored at −80°C until subsequent analysis. The paraffin wax embedded samples were sectioned (5 µM) and stained with haematoxylin and eosin (H&E), and Masson Trichrome (MT) using standardised histological techniques.

### 2.3 Histological Quantification of Healing Dynamics

The stained histology slides (H&E and MT) were imaged using the Axio Observer Microscope (Zeiss) and the Grundium Ocus 40 tile scanner (Grundium, Finland) at ×10 magnification. Healing dynamics of the wound sections were quantified using a standardized histology scoring system—SPOT skin wound score [25]. The following parameters were quantified: re‐epithelization (%), epithelial thickness index (ETI), keratinization, granulation tissue thickness (µm), scar elevation index (SEI), and remodelling. Remodelling included the assessment of collagen deposition, dermal white adipose tissue (dWAT) regeneration, formation of skin appendages, and repair of *panniculus carnosus* muscle. The overall histology score reported (SPOT skin wound score) is a weighted compilation of all the parameters assessed as indicated in Table 1. Additional parameters that were assessed included cellular infiltration (number of nuclei per surface area) in the wound edge and wound area at the respective time points.

**Table 1:** Histology: SPOT skin wound score.

Parameters	Scoring criteria			Assigned score		Parameter specification	
**Re‐epithelization**	%Re‐epithelization = (distance of axis covered by epithelium/distance of minor axis between original wound edges) × 100			2		Complete (95–100%)	
				1		Partial (< 95%)	
				0		None (0%)	
**Epidermal thickness index**	ETI = (average thickness of epidermis in wound area/average thickness of epidermis in uninjured skin) × 100			2		Normal (95–105%)	
				1		Hypertrophy ( > 105%)	
				0		Hypoplasia ( < 95%)	
**Keratinization**	Loosely attached or lost layers OR thick parakeratotic stratum corneum			2		Yes	
				0		No	
**Granulation tissue**	Visual inspection and absolute measure (µm)			2		Intact dermis – no granular infiltrates consistent with healing	
				1		Thick granulation tissue (> 100 µm)	
				0		Thin granulation (< 100 µm)	
**Remodelling**	1 = dWAT 2 = skin appendages 3 = collagen deposition 4 = panniculus carnosus regeneration			2		Complete (1,2,3,4)	
				1		Partial (1,2,3)	
				0		None	
**Scar elevation index**	SEI = (average dermis thickness in wound area/average dermis thickness in uninjures skin) × 100			2		Normal (95–105%)	
				1		Hypertrophied (> 105%)	
				0		Hypoplasia (< 95%)	

	**Total Score**			**12**			
**Histology score**		0	1–3	4–7	8–11		12
Stage of healing		No signs of healing	Inflammation	Proliferation	Remodelling		Completely healed

*Note:* This table is based on the minimum recommended criteria for assessing wound healing dynamics using the SPOT skin wound score [3].

### 2.4 Immunohistochemistry

The expression of MPO (neutrophil marker) and tumour necrosis factor α (TNF‐α) (pro‐inflammatory cytokine) were assessed using standardised immunohistochemistry techniques with endogenous tissue background controls and negative antibody controls used to eliminate false positives. The tissue sections were stained with the respective primary antibodies (MPO, QU087717; TNF‐α, Sc52746) (Abcam, Santa Cruz), followed by the fluorescent conjugated secondary antibodies (MPO: Goat anti‐mouse IgG AF488 secondary antibody, ab150113; TNF‐α: Donkey anti‐mouse AF488, ab150109). The sections were counterstained with NucBlue Live Ready Probes (Invitrogen, Thermo Fisher Scientific, USA) and imaged using the Nikon Eclipse TI2 fluorescent microscope at 10× magnification. Mouse spleen served as the positive control, while phosphate buffered saline was added instead of the primary antibody to serve as the negative control. Images of the wound edges (4 fields of view) and wound area (×4 fields of view) were captured, and the mean fluorescent intensity with background subtraction quantified using the Fiji Image J software.

### 2.5 ELISAs: Eicosanoids and MMP‐9 Expression

Protein was extracted from the snap frozen wound tissue samples by placing the samples in Tris buffer (0.1 M; 1%SDS, pH8) followed by homogenization and sonication at 4°C. This was followed by centrifugation and the supernatant used for subsequent analysis. Protein concentrations were quantified using a nanodrop (Jenway 7415 Nano Scanning Micro‐Volume Spectrophotometer, Cole Palmer, USA) at 260/280 nm and the concentration normalized to 16 µg. The concentration of thromboxane A2 (TxA2) (E‐EL‐0057, Elabscience), leukotriene B4 (LTB4) (E‐EL‐0061, Elabscience), prostaglandin E2 (PGE2) (E‐EL‐0034, Elabscience), protectin D1 (PD1) (ELK0497, ELK Biotechnology), maresin 1 (MaR1) (ELK0496, ELK Biotechnology), resolvin D1 (RvD1) (ELK0494, ELK Biotechnology), resolvin E1 (RvE1) (ELK0495, ELK Biotechnology) and matrix metalloproteinase ‐9 (MMP‐9) was assessed using either competitive or sandwiched based ELISA kits according to the manufacturer's instructions. The absorbance was read at 450 nm using a spectrophotometer (Multiskan GO, Thermo Fisher Scientific, USA). The inter‐assay variability (CV) of the respective ELISA kits were 5% with a recovery of 80–120%.

## 3 Statistical Analysis

Statistical analysis was performed using GraphPad Prism version 10 (GraphPad Software Inc., San Diego, USA). All analysis was performed in triplicate with outliers (if any) determined using ROUT (1%). All values are presented as either mean ± SE or percentage (%) (*n*/*N*). Two‐way ANOVA with Tukey post hoc test or Mixed effects ANOVA with Sidak's multiple comparisons test was used to determine the effect of time, group, and time x group. Simple linear regression analysis was performed to determine significant associations between variables. The threshold level for significance was accepted at *p* < 0.05.

## 4 Results

### 4.1 Diabetic Wounds Exhibit Delayed Superficial Wound Closure and Re‐Epithelisation

**Figure 1:**
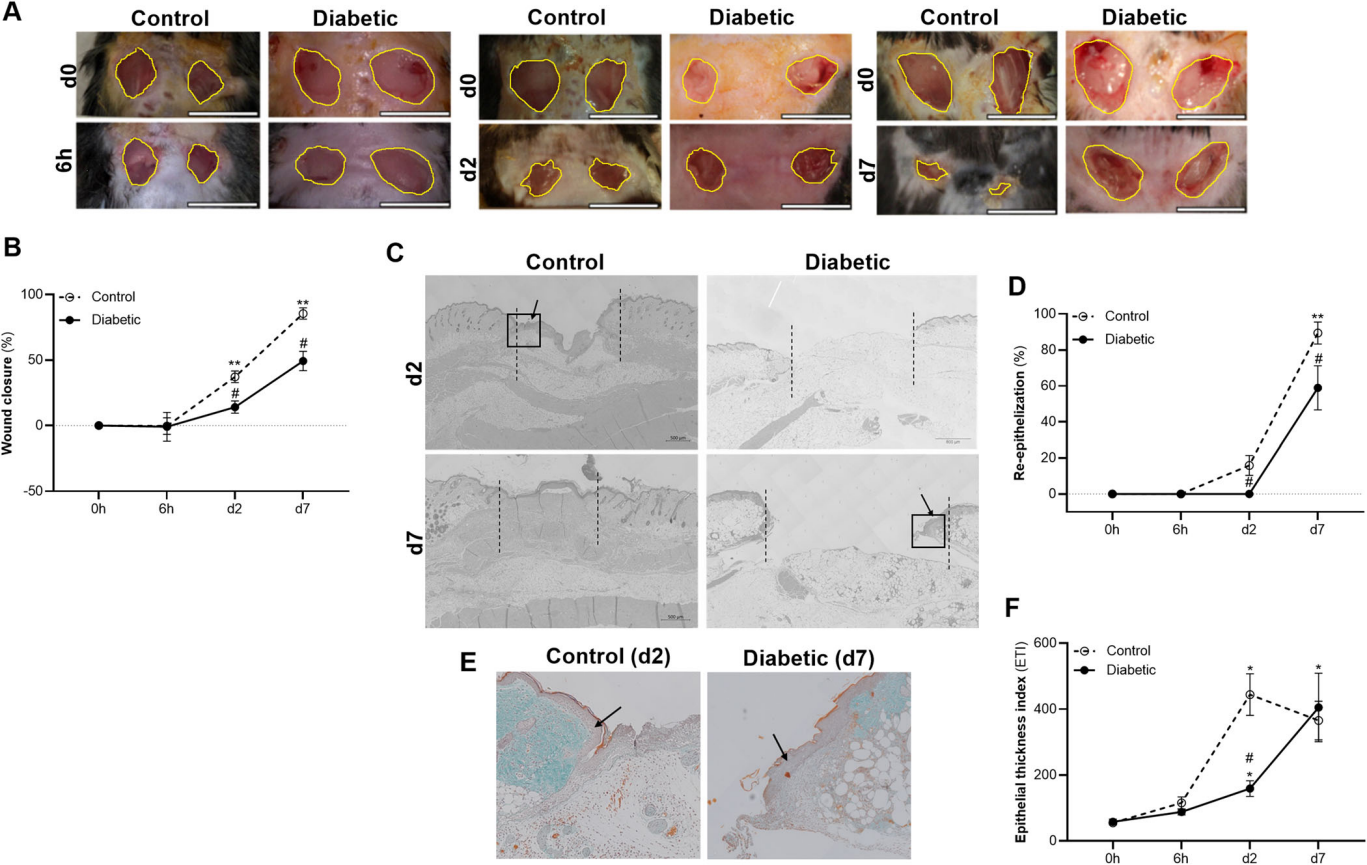
Superficial wound closure. (A) Representative images illustrating wound surface area at 6 h, day2 and day 7 post wounding in the control (*n* = 6) and diabetic (*n* = 6) animals respectively. (B) The percentage wound closure over time (%). (C) Representative histology images (Masson's Trichrome) illustrating the wound area (dotted lines) within the respective groups on days 2 and 7 post wounding. (D) Quantification of the percentage re‐epithelization (%) (mean ± SE) (distance of axis covered by epithelium in wound area/distance between original wound edges × 100). (E) Higher magnification of the wound edges (black squares) on histology images illustrating epithelial hypertrophy (black arrows) in the wound edges on day 2 in the control group and on day 7 in the diabetic group. (F) Quantification of the epithelial thickness index (ETI) (mean ± SE) (average thickness of the epidermis in wound area/average thickness of the epidermis in uninjured skin). Statistical analysis: Analysis of Variance (ANOVA) with mixed‐effects multiple comparisons (Sidak correction). **p < 0.01 indicate significant effect of time. #*p* < 0.05 indicate significant difference between groups.

Superficial wound closure over time was quantified based on macroscopic wound surface area as indicated in Figure 1A. Wound closure was evident from day 2 onwards in the control animals, whereas the diabetic wounds remained open for the duration of the study. On days 2 and 7 post wounding there was thus significantly greater (*p* < 0.01) wound closure in the control (day 2 37 ± 14%; day 7 85 ± 14%) compared to the diabetic (day 2 14 ± 15%; day 7 49 ± 23%) animals (Figure 1B). In agreement, on histological level the control wounds showed signs of re‐epithelisation on day 2 (15.8 ± 16.5%), with 89 ± 19% of the initial wound areas re‐epithelized on day 7 post wounding. In contrast, no signs of re‐epithelization were evident on day 2 in the diabetic wounds with only partial re‐epithelisation (58.8 ± 34.7%) evident on day 7 post wounding (Figure 1C‐D). Comparison of the extent of re‐epithelisation (%) between the respective groups at the different time points confirmed a significant (*p* < 0.01) delay in re‐epithelisation in the diabetic wounds (Figure 1D). In agreement, thickening of the epithelial layer in the wound edges peaked on day 2 post wounding in the control wounds (ETI day 2: control 444 ± 188; diabetic 159 ± 62) (*p* < 0.01), whereas epithelial hypertrophy in the diabetic wound edges only became evident on day 7 (ETI day 7: control 365 ± 185; diabetic 405 ± 294) (*p* < 0.01) (Figure 1E‐F). Taken together this data confirms that superficial wound closure was significantly delayed in the diabetic animals.

### 4.2 Early‐Hours Post Wounding: Expression of MMP‐9 and Eicosanoids

**Figure 2:**
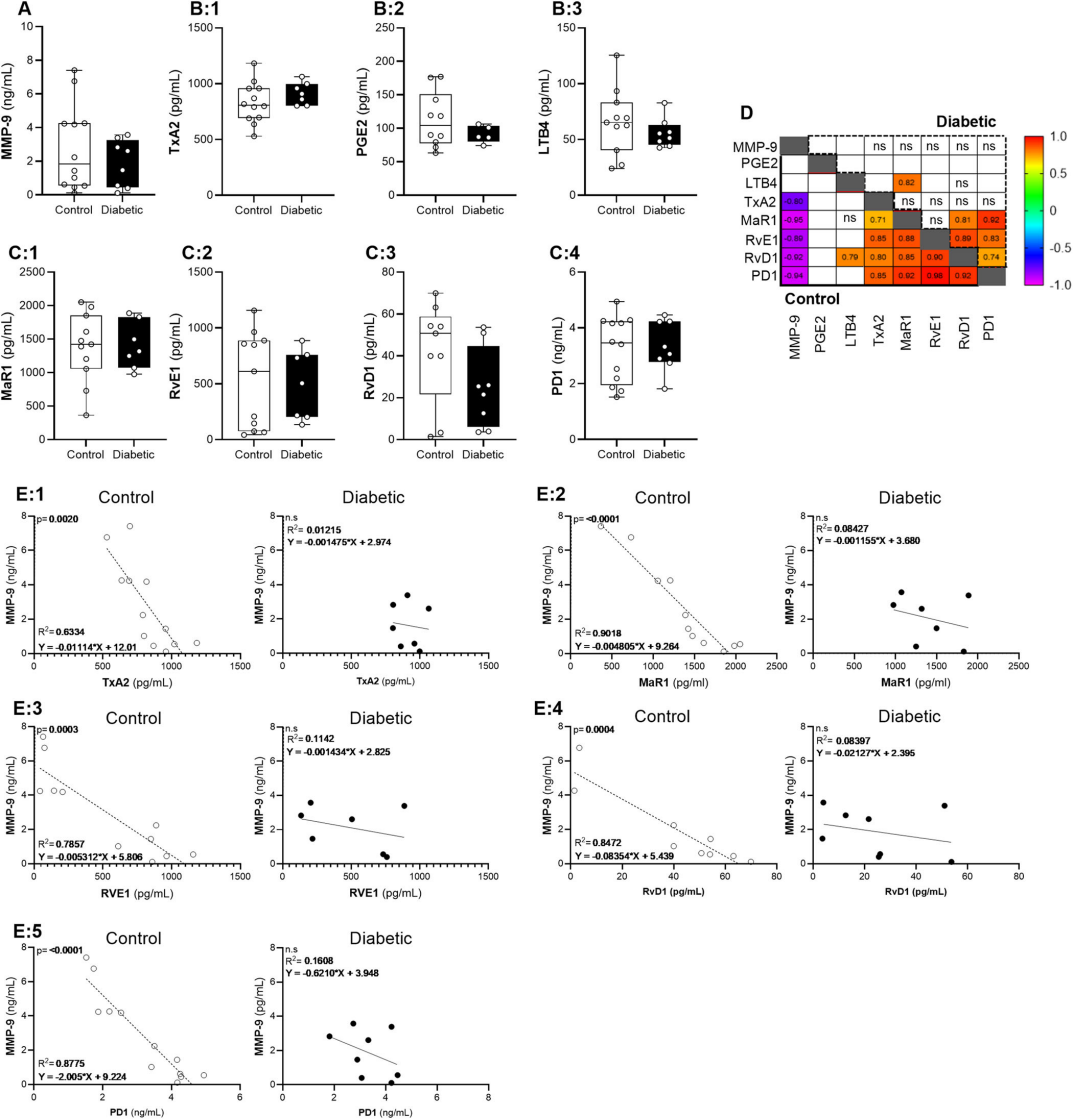
Early‐hours post wounding: MMP‐9 and Eicosanoids. (A) MMP‐9 expression within the wounded tissue at 6 h post wounding in the control (*n* = 6) and diabetic (*n* = 6) wounds. (B) The expression of the pro‐inflammatory lipid mediators, TxA2 (B.1), PGE2 (B.2) and LTB4 (B.3) as well as the expression of the (C) Specialized pro‐resolving lipid mediators MaR1 (C.1), RvE1 (C.2), RvD1 (C.3) and PD1 (C.4) within the wounded tissue at 6 h post wounding. Statistical analysis: Unpaired *t*‐test. (D) Correlation matrix illustrating the significant R2‐values within the control and diabetic group respectively. (E) Spearman's correlation analysis indicating an inverse association between MMP‐9 and TxA2 (E.1), MaR1 (E.2), RvE1 (E.3), RvD1 (E.4) and PD1 (E.5) within the control groups, whereas no correlation was evident in the diabetic group.

In the early stages post wounding (6 h), the expression of MMP‐9 was variable with no difference detected between groups (control 3 ± 1; diabetic 2 ± 0.5 ng/mL) (Figure 2A). Similarly, no difference was detected in the pro‐inflammatory lipid mediators (TxA2: control 829 ± 52; diabetic 914 ± 37 pg/mL, PGE2: control 113 ± 13; diabetic 91 ± 6 pg/mL and LTB4: control 66 ± 9; diabetic 56 ± 5 pg/mL) (Figure 2B1‐3) or specialized pro‐resolving mediators (MaR1: control 1377 ± 156; diabetic 1405 ± 133 pg/mL, RvE1: control 534 ± 129; diabetic 492 ± 117 pg/mL, RvD1: control 42 ± 8; diabetic 25 ± 7 pg/mL, PD1: control 3 ± 0.3; diabetic 3 ± 0.3 ng/mL) (Figure 2C1‐4) between groups. Correlation analysis did however illustrate significant associations between the expression of the various lipid mediators with significant R^2^ values indicated in Figure 2D. A significant positive correlation was evident between the pro‐inflammatory mediator, TxA2 and the pro‐resolving mediators (MaR1, RvE1, RvD1, PD1) in the control group but not in the diabetic group (Figure 2D). Similarly, a significant inverse correlation (*p* < 0.05) was furthermore observed between MMP‐9 and TxA2, MaR1, RvE1, RvD1, PD1 respectively in the control group, this association was however not evident in the diabetic group (Figure 2D‐E).

### 4.3 Diminished Cellular Infiltration in Diabetic Wounds Coincided With a Lacking TNF‐α Response and Delayed Expression of MPO

**Figure 3:**
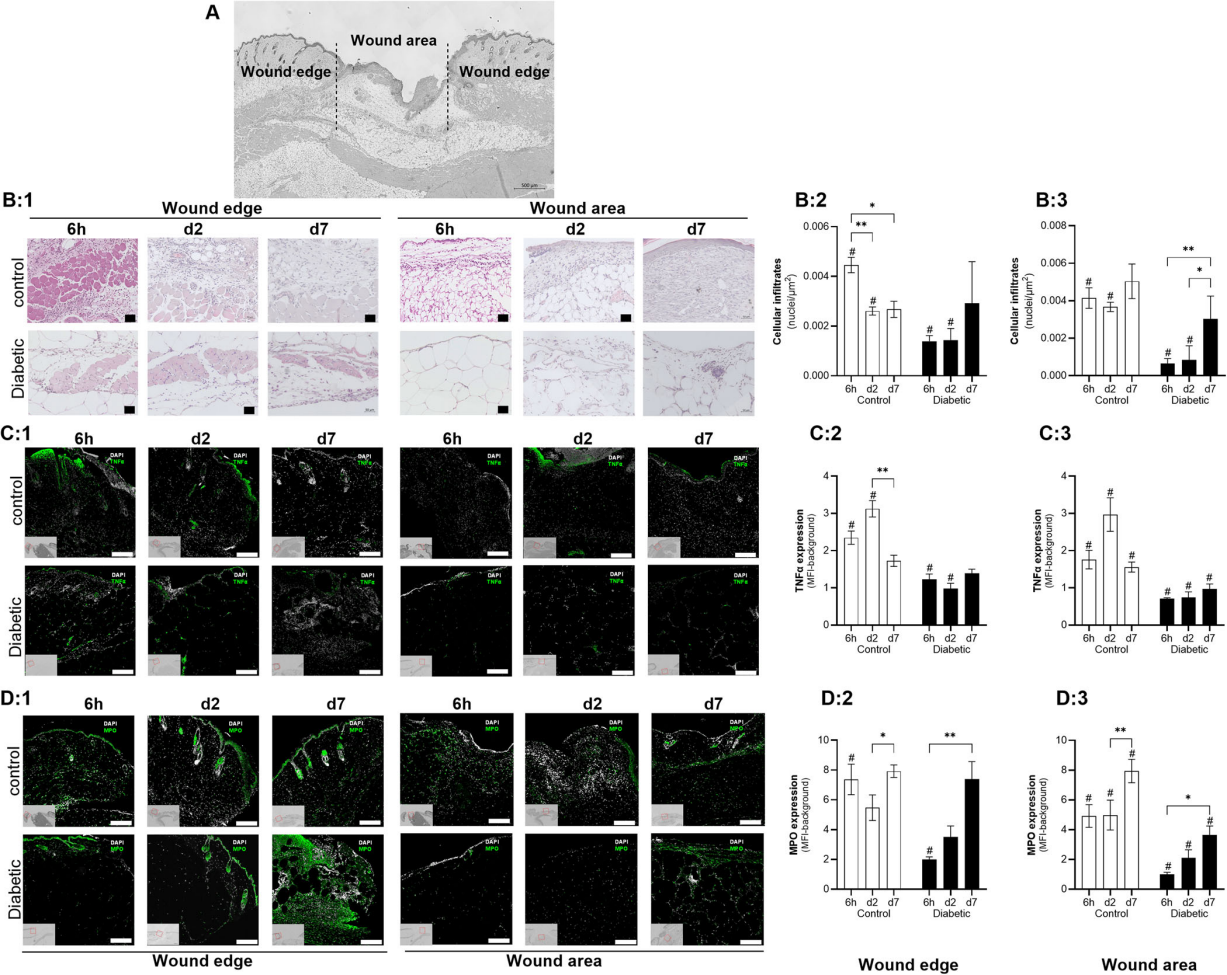
Cellular infiltration, TNF‐α and MPO expression. (A) Representative H&E histology image illustrating the wound area (dotted lines) and wound edges on either side of the wound area. (B1) Representative H&E images (field of view) of the wound edges and wound areas at the respective time points. (B2‐3) Quantification of cellular infiltrates (nuclei/µm^2^) in the wound edges (B2) and wound area (B3) within the control (*n* = 6) and diabetic (*n* = 6) groups over time. (C1) Representative TNF‐α immunohistochemistry images (field of view) of the wound edges and wound areas at the respective time points. (C2‐3) Quantification of TNF‐α (mean fluorescent intensity) in the wound edges (C2) and wound area (C3) within the control (*n* = 6) and diabetic (*n* = 6) groups over time. (D1) Representative MPO immunohistochemistry images (field of view) of the wound edges and wound areas at the respective time points. (D2‐3) Quantification of MPO (mean fluorescent intensity) in the wound edges (C2) and wound area (C3) within the control (*n* = 6) and diabetic (*n* = 6) groups over time.Statistical analysis: Analysis of Variance (ANOVA) with mixed‐effects multiple comparisons (Sidak correction). *p < 0.05, **p < 0.01, indicate significant effect of time and #*p* < 0.01 indicate significant difference between groups at the same time point.

Histological analysis of the wound edges (×4 fields of view) and wound area (×4 fields of view) (Figure 3A) illustrated that cellular infiltration into the wound edges peaked at 6 h post wounding and remained elevated on days 2 and 7 in the control wounds, whereas signs of cellular infiltration in the diabetic wounds only became evident on day 7 post wounding (Figure 3B1‐3). Quantification of cellular infiltration indicated fivefold (6 h, day2) and 1.6‐fold (day 7) less nuclei per µm^2^ in diabetic compared to control wounds (Figure 3B1‐3). This observation was confirmed by immunohistochemical staining assessing the expression of the pro‐inflammatory cytokine, TNF‐α and the neutrophil marker, MPO within the wound edges and wound areas. In the control wounds a peak in TNF‐α expression was evident on day 2 post wounding in both the wound edge as well as in the wound bed (wound area), whereas this was not evident in the diabetic wounds (Figure 3C1‐3). Quantification indicated that at 6 h post wounding, there was already significantly higher levels of TNF‐α present in the control compared to diabetic wounds in the wound edge (*p* < 0.001), and wound bed (*p* < 0.05) (Figure 3C2‐3). In the diabetic wounds, no increase in the expression of TNF‐α on tissue level was detected at any of the time points assessed (Figure 3C2‐3).

Notable differences in MPO expression as indicator of neutrophil phagocytic activity were also observed between the control and diabetic wounds at the different timepoints (Figure 3D1‐3). At 6 h post wounding, the control wounds expressed significantly higher MPO levels compared to the diabetic wounds in the wound edges (*p* < 0.001), and wound area (*p* < 0.01) (Figure 3D2‐3). In the control wounds, MPO expression levels remained elevated with a secondary peak evident on day 7 post wounding in both the wound edge and wound area (Figure 3D2‐3). A significant increase in MPO expression was however only evident on day 7 post wounding in the diabetic animals (Figure 3D2‐3).

### 4.4 Wound Healing Dynamics: Histology Score

**Figure 4:**
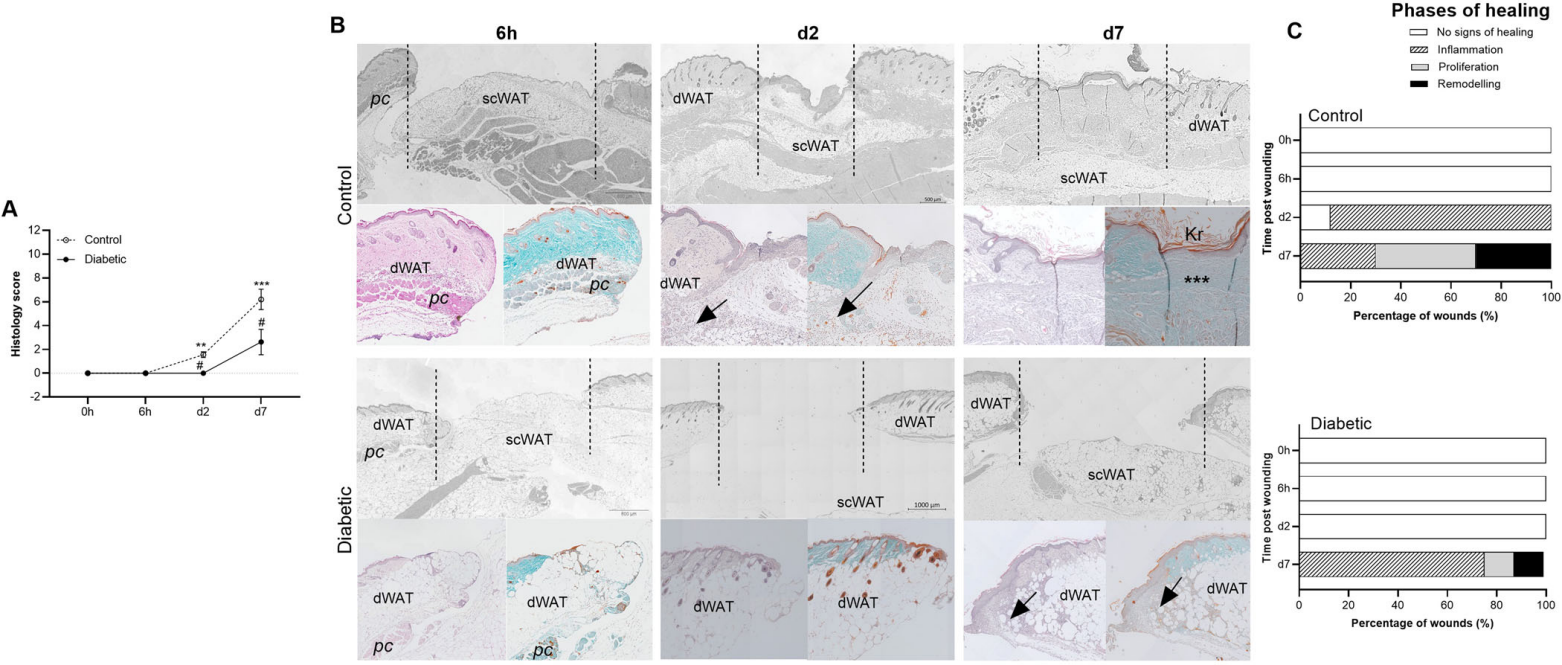
Comparison of the progression through the phases of healing between groups. (A) Quantification of the overall histology score over time between the control (*n* = 6) and diabetic (*n* = 6) groups. Statistical analysis: Analysis of Variance (ANOVA) with mixed‐effects multiple comparisons (Sidak correction). *p < 0.05, **p < 0.01, indicate significant effect of time and #*p* < 0.01 indicate significant difference between groups at the same time point. (B) Representative histology images (H&E, MT) illustrating the wound area (dotted lines). dWAT – dermal white adipose tissue, scWAT – subcutaneous white adipose tissue, pc – panniculus carnosus muscle, Kr – keratinization, *** ‐ collagen deposition. (C) The percentage of wounds (%) within each specific phase of healing over time for the control (*n* = 6) and diabetic (*n* = 6) group, respectively.

Wound healing dynamics and the phases of healing was assessed histologically and quantified using the SPOT skin wound score. During the first 6 h post wounding, no signs of healing was evident on histological level (histology score: 0) in the control and diabetic wounds (Figure 4A). On day 2 post wounding, the control wounds were in the inflammatory stage (histology score: 1.56 ± 0.73) and on day 7 had transitioned into the proliferative and remodelling stages of healing (histology score 6.2 ± 2.7) (Figure 4A‐C). In the diabetic wounds no signs of healing (histology score: 0) were evident for the first 2 days post wounding, with diabetic wounds only entering the inflammatory stage (histology score: 2.6 ± 3.0) on day 7 post wounding (Figure 4A‐C). A breakdown of the SPOT skin wound score and detailed assessment of the histological parameters revealed noticeable differences between the respective groups (Figure 4B).

Keratinization was not observed in the wound tissue before day 7 in either the control or diabetic wounds. On day 7, most control wounds (70%, 7/10) (n/N) exhibited loose layers of keratin with some wounds exhibiting a thick parakeratotic stratum corneum (Figure 4B). Whilst in the diabetic group, the wounds remained open (75%, 6/8) (n/N) and had no keratinization. Granulation tissue formation was not evident in the wounds during the first 6 h post wounding. In the control group, on day 2, thick granulation tissue (> 100 µm) had already formed in 77% (7/9) (n/N) of the wounds. The formation of granulation tissue was delayed in the diabetic wounds, with thin granulation tissue (< 100 µm) only evident in the wound edges (7/7) (n/N) on day 7 post wounding (Figure 4B).

On day 7, only 1/10 (n/N) animals in the control group exhibited dermal hypertrophy indicative of scar formation, whereas the remainder of wounds had either an intact dermal layer consistent with complete healing (2/10) (n/N) or had dermal hypoplasia and were still in the proliferative and remodelling stages of healing (7/10) (n/N). At the same time point, very little to no restoration of the wound bed was evident in the diabetic wounds (Figure 4B).

## 5 Discussion

Wound healing is a tightly regulated and coordinated process that involves multiple cell types and molecular pathways [11]. Disruption of various processes in the healing cascade due to the multifactorial pathological changes within the diabetic microenvironment prevents progression through the phases of healing leading to wound deterioration. The persistent inflammatory characteristics and molecular dysregulation evident in chronic diabetic wounds that have failed to heal have been frequently described in diabetic patients [26]. Several animal‐based studies have, however, illustrated an inadequate acute inflammatory response upon tissue injury [27, 28, 29]. Yet, there is little to no information available on the spatial dynamics and eicosanoid dysregulation during the first few hours post wounding and its impact on the progression of healing.

The current study therefore investigated the expression of pro‐inflammatory and pro‐resolving mediators during the early stages post injury and compared the timeframe for transitioning through the phases of healing on histological level in acute and diabetic wounds. This study illustrated that during the first few hours post injury an inverse relationship exists between the expression of MMP‐9 and eicosanoids under normal circumstances but that this association is lost in diabetic wounds. Diminished cellular infiltration in diabetic wounds furthermore coincided with a significant delay in the expression of early inflammatory markers, TNF‐α (pro‐inflammatory cytokine) and MPO (neutrophil marker). Of note, a significant difference in the expression of TNF‐α and MPO was observed as early as 6 h post wounding, with histology parameters supporting the notion that there is a significant delay in the onset of the acute inflammatory response in diabetic wounds. This is consistent with the observations of other recent reports from animal studies showing reduced expression of pro‐inflammatory cytokines during the early stages post injury and a delayed influx of immune cells into diabetic wounds [27, 30, 31]. Given the complexity and integrated nature of the healing process failure to initiate the acute inflammatory response could adversely affect transitioning into the proliferative and remodelling stages of healing.

Eicosanoids play a crucial role in the wound healing process, with well described roles in initiating, regulating and resolving inflammation [12]. Despite research on systemic eicosanoid levels in diabetic individuals and infection models, the role of eicosanoids in diabetic wounds remains poorly understood [1]. This is the first study to report on eicosanoid levels as early as 6 h post injury during the early inflammatory initiation phase. PGE2 is a complex lipid mediator with diverse physiological outputs depending on its interaction via four different isoforms of its G protein coupled receptors. Consistent with other reports, PGE2 was produced following injury [32], in this study, no difference was however detected between groups. Similarly, TxA2 and LTB4 are arachidonic metabolites that play a key role in the initial stages of wound healing. Following injury, activated platelets produce TxA2, which further activates platelets, facilitate platelet aggregation, neutrophil recruitment and activation, and promote M1 macrophage phenotype [33, 34]. TxA2 is thought to activate neutrophils through receptor binding and by doing so promotes MPO production and the formation of NETs, which are essential antimicrobial functions to prevent infection [35, 36]. Similarly, LTB4 promotes and amplifies the recruitment of neutrophils to the injured or infected site and plays a role in their activation, phagocytosis and cytokine production [37]. In the current study, despite similar expression levels of eicosanoids at the 6 h time point a strong correlation between TxA2 and MPO (neutrophil marker) was only observed in the control wounds, with very little to no cellular infiltration evident in the diabetic wounds, indicative of delayed neutrophil trafficking. Suggesting that the immune cells are unresponsive to the initial eicosanoid expression in the wound tissue. In support of this notion, Roy et al. [27] previously indicated that hyperglycaemia‐induced defects in the FPR receptor on neutrophils reduces the chemotactic response of these cells. Similarly, several other studies have implicated the high concentrations of AGEs, a common feature of diabetes, in impaired neutrophil migration and aggregation, and can impair the responsiveness of macrophages to bacterial LPS stimulation [38, 39, 40]. Whilst others have shown that the continuous activation of immune cells in the diabetic environment, results in immune exhaustion or senescence, whereby immune cells fail to respond to acute stimuli and exhibit dysfunctional behaviour, that is, immune cells do not function at their optimal level [24, 41]. A comprehensive review by our group describes the mechanistic dysregulation of innate immune cells in metabolic disease [24]. The delay in immune cell infiltration, increases the risk of wound infection and bacterial colonization (lack of first line immune defence), a hallmark of diabetic wounds, and prolong the wound healing process [42]. Indeed, Mahmud et al. [43] suggested that stimulating the innate immune system using immunomodulators to increase the acute inflammatory response can improve infection control and stimulate healing in surgical wounds. It is thus essential to improve our understanding of the underlying mechanisms associated with the delayed acute inflammatory response in diabetic wounds.

MMP‐9 is a well‐known matrix metalloproteinase with a noteworthy role in wound healing. Clinical studies have consistently shown that during the later stages, diabetic wounds exhibit increased levels of MMP‐9, which is widely recognized as a key contributor to impaired wound healing and delayed recovery [44, 45]. On the other hand, mice lacking MMP‐9 displays delayed healing and disordered collagen fibrils [46], with numerous studies showing that MMP‐9 also plays an important immunomodulatory role [45, 47]. In the current study, additional analysis revealed a strong inverse relationship between MMP‐9 and several of the eicosanoids (TxA2, MaR1, RvE1, RvD1, PD1). The association between MMP‐9 and TxA2 has been shown previously with MMP‐9 suppressing platelet aggregation by blocking the production of TxA2 [48]. Strong inverse correlations were also observed between MMP‐9 and the pro‐resolving lipid mediators (MAR1, RvE1, RvD1 and PD1). Pro‐resolving mediators are essential for progression through the phases of healing by reducing inflammation and promoting transitioning into the proliferative stage [12]. It is thus expected that in the early stages, whilst the acute immune response is being initiated, the expression of these pro‐resolving mediators should be suppressed. Studies have, however, revealed that an active and highly coordinated resolution program is already launched in the early stages of inflammation, within the first few hours of its onset [49]. In agreement, strong positive correlations between TxA2 and the resolution eicosanoids (known to inhibit neutrophil infiltration and cytokine production) were evident in the early hours post wounding. These correlations highlight the possible regulatory feature of TxA2 in wound healing and is in agreement with previous studies illustrating that in addition to its role in hemostasis, TXA2 also plays a role in fibroblast contractility during the later stages of healing [50, 51]. These associations were clearly evident in the control wounds, but not in the diabetic wounds and could be indicative of a possible dysregulation or be a consequence of the lack of immune cell infiltration and warrants further investigation. A limitation of the current study was only assessing the expression of eicosanoids at the 6 h time point, nonetheless the cellular infiltration and histology data suggest that the immune response was delayed by a week (7days) in the diabetic wounds. These observations imply that the immune cells are unresponsive to the initial eicosanoid expression in the diabetic wound tissue and that early intervention is required to stimulate the acute response following injury. Indeed, a previous study by [52] illustrated that topical application of bacterial LPS upregulated the expression of pro‐inflammatory cytokines, increased macrophage infiltration and growth factor release and promoted wound healing.

## 6 Conclusion

This study illustrated a delayed onset immune response in diabetic wounds which could be detrimental to healing outcomes. The data furthermore illustrated that the association between MMP‐9 and eicosanoid signalling is lost in diabetic wounds during the first few hours post injury. The clinical significance of this finding is still unclear and could be indicative of a possible dysregulation or be a consequence of the lack of immune cell infiltration. A limitation of the current study is the short 7‐day observational period providing a snapshot of events, more in‐depth analysis over the full healing period is required to fully understand the mechanisms underlying the dysregulated immune responses and its impact on the transitioning through the phases of healing. Future studies should investigate the indirect consequences of immune dysfunction in diabetic wounds to identify possible novel therapeutic strategies focussed on stimulating the acute immune response.

## Author Contributions

**Priyanka Dhanraj:** data curation; formal analysis; investigation; project administration; visualization; writing–original draft. **Kiara Boodhoo:** data curation; investigation; methodology; writing–review & editing. **Mari van de Vyver:** conceptualization; funding acquisition; methodology; supervision; writing–review & editing.

## Conflicts of Interest

The authors declare no conflicts of interest.

## Data Availability

Data is available upon request from authors.
